# A multi-color video-ophthalmoscopes allows to measure the spectral distribution of light absorption of blood in the human retina

**DOI:** 10.3389/fmed.2023.1125154

**Published:** 2023-03-16

**Authors:** Ralf-Peter Tornow, Jan Odstrcilik, Radim Kolar

**Affiliations:** ^1^Department of Ophthalmology, Friedrich-Alexander Universität Erlangen-Nürnberg, Erlangen, Germany; ^2^Department of Biomedical Engineering, Faculty of Electrical Engineering and Communication, Brno University of Technology, Brno, Czechia

**Keywords:** blood light absorption, photoplethysmography, video ophthalmoscopy, retina, multi-color imaging, retinal imaging

## Abstract

Based on our previously developed mono-color video-ophthalmoscope a multi-color video-ophthalmoscope was developed. Using narrow band transmission filters, this instrument allows to measure the pulsatile cardiac cycle induced blood volume changes in the human retina for any wavelength in the sensitivity range of the used CMOS-camera. In this key experiment, video sequences (8 s, 25 fps, 200 frames) of the optic nerve head (ONH) were acquire for seven wavelengths between 475 nm and 677 nm one after the other. After image registration of all frames of each video sequence (to compensate for eye movements) and trend correction (to compensate for slow intensity changes), the amplitude of the cardiac cycle induced light intensity changes (pulsatile absorption amplitude PAA) can be calculated for all seven wavelengths. The results confirmed that the spectral distribution of PAA (λ) follows the distribution of the light absorption of blood. The measured values correspond to the absorption of a thin blood layer of about 0.5 μm thickness.

## 1. Introduction

Retinal imaging is an important tool in both clinical routine and research. Compared to single shot still fundus cameras, a video-ophthalmoscope allows to assess even dynamic processes like changing blood volume (1), vessel movements, spontaneous venous pulsation (2) and fixation behavior (3). So far, the illuminating wavelength of our developed mono-color video-ophthalmoscope was restricted to 577 nm of a narrow band LED. The key experiment described here aims to show that it is relatively easy to expand this instrument into a multi-color video-ophthalmoscope that allows retinal video sequences to be recorded at any wavelength in the sensitivity range of the CMOS or CCD camera. It is important to optimize the wavelength for the clinical application and to confirm that the pulsatile light absorption is caused by the absorption of changing blood volume.

Multi-color video-ophthalmoscope means that video sequences can be acquired with different wavelengths of the illuminating light. At the present state of development of our video-ophthalmoscope, the video-sequences for different wavelengths have to be acquired successively, but parallel acquisition will also be possible. Commercial fundus cameras use RGB (red, green, and blue) CCD or CMOS sensors to acquire color fundus images. This is also a multi-color technique, however, the spectral distribution of the three color bands is relatively broad (typically in the range of 100 nm) with overlapping ranges and the wavelength cannot be changed by the user. On the other hand, the different colors are acquired simultaneously.

Some modifications of commercial fundus cameras to get narrow band multi-color fundus images are described in the literature. Mordant et al. (4) describes the use of a modified fundus camera to obtain sequential hyperspectral images for oximetry within retinal vessels. Al Zoubi et al. (5) uses multispectral imaging to record the optic disk reflectance at wavelengths 522 nm, 548 nm, 555 nm, 586 nm, and 610 nm to calculate hemoglobin concentration and oxygenation (SO_2_). Toslak et al. (6) uses four narrowband (17 nm–60 nm) LEDs, (530 nm, 625 nm, 780 nm, and 970 nm) for multispectral imaging with a contact-mode ultra-widefield system with trans-pars-planar illumination. In all described modifications, the images are acquired sequentially. This has the advantage that a monochrome sensor can be used. The light sensitivity of monochromatic sensors is higher than that of RGB sensors. This is important to keep the light intensity below the maximum permissible exposure level of the eye when acquiring video sequences. In our instrument, we use a monochrome CMOS sensor with high light sensitivity and sequential acquisition of the different wavelengths.

Applying the multi-color video-ophthalmoscope, we show that the amplitude of the cardiac cycle-induced light absorption in the retina follows the spectral distribution of the light absorption of blood. Changing blood volume results in changing light absorption of the retina. Higher blood volume results in higher absorption and hence lower light intensity at the image detector and vice versa. The highest change in light absorption during one heart beat (pulsatile absorption amplitude, PAA) can be calculated from the time course of the pulsatile change in intensity (7). In the present experiment, we evaluated the PAA in small areas of the optic nerve head. The PAA is compared with the light absorption of blood.

The aim of this paper is to show in a key experiment that the mono-color video-ophthalmoscope can be extend to a multi-color video-ophthalmoscope and that the measured spectral distribution of PAA(λ) represents the spectral distribution of the light absorption of a thin blood layer. These findings can be used for further optimization of the wavelength of a mono-color-video-ophthalmoscope for different clinical applications.

## 2. Materials and methods

In this section, the instrument, the calculation of the final photo-plethysmography signal I_pleth_ (*n*) from the video sequences, the calculation of the pulsatile absorption amplitude (PAA) and the absorption of a thin blood layer are described.

### 2.1. The instrument

**Figure 1:**
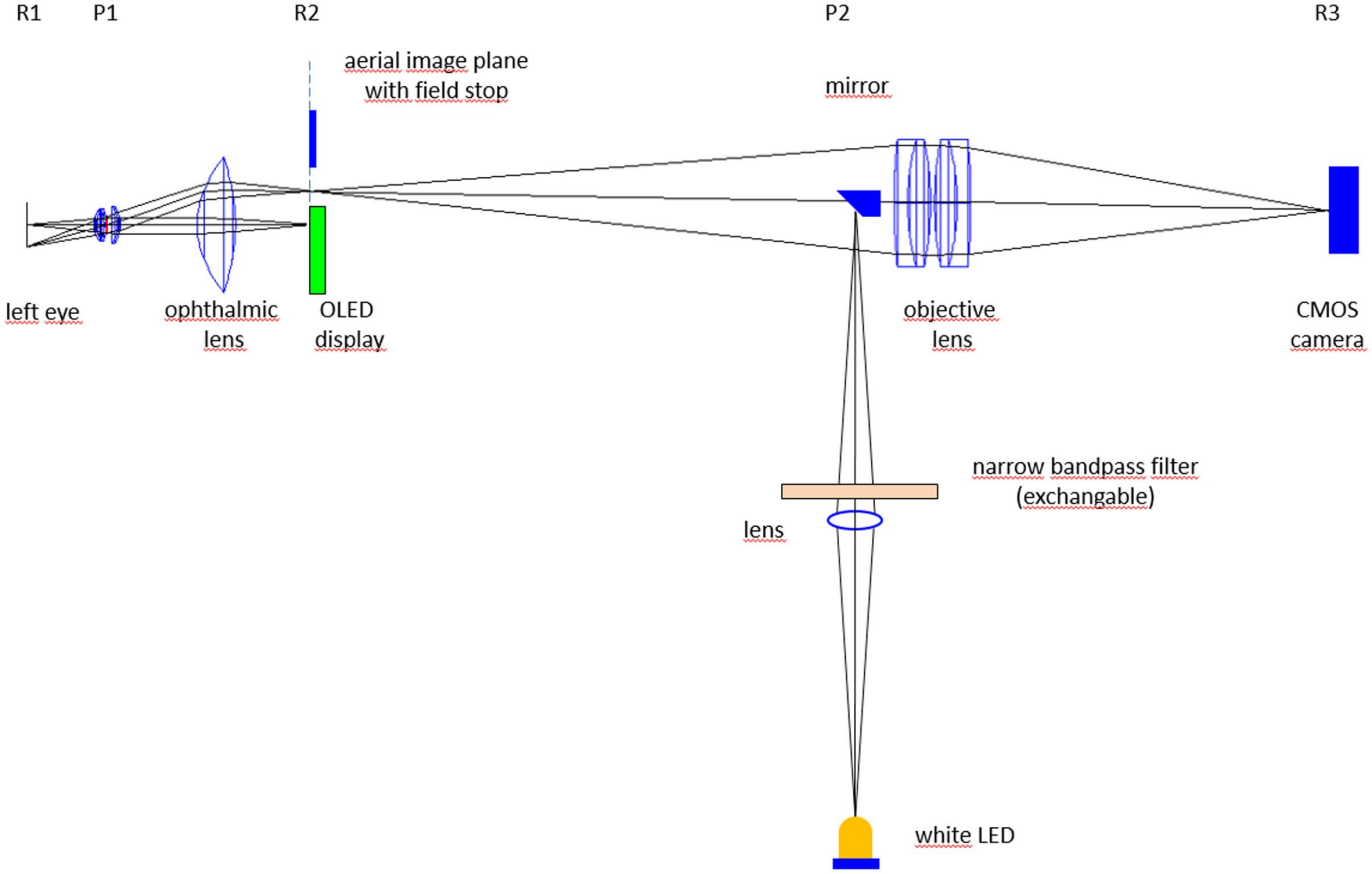
Experimental set-up of the multi-color video-ophthalmoscope. Rx and Px represent retinal (R) and pupil planes (P).

The multi-color video-ophthalmoscope (Figure 1) consists of a Volk optical lens (40D) that projects an image of the retina in the aerial image plane (for details see Refs. (7, 8)). This aerial image is projected by an objective (two achromatic lenses) to a CMOS camera with high light sensitivity. An OLED display in the aerial image plane is used as fixation target to reduce eye movements of the subject during video acquisition. Compared to the video-ophthalmoscope described in Tornow et al. (7), here the illuminating LED is replaced by a 45 deg. mirror (diameter 5 mm) that is placed directly in the beam path of the ophthalmoscope at the position of an image of the pupil plane (P2 in Figure 1). A white LED placed outside the optical axis of the video-ophthalmoscope is imaged on to the 45 deg. mirror. By inserting narrow bandpass filters between the white LED and the 45 deg. mirror, different wavelengths can be selected for illumination.

For each narrow bandpass filter, the intensity in the pupil plane (P1 in Figure 1) was set to 12 μW by adjusting the LED current and if necessary by inserting neutral density filters. This intensity in the pupil plane results in a retinal illumination of 30 μW/cm^2^ (intensity of 12 μW divided by exposed area of 0.4 cm^2^) what is significantly below the maximum permissible exposure level (MPE) of 220 μW/ cm^2^ for long exposure (9). To compensate for the different reflection of the ocular fundus for the different wavelengths, the gain of the camera was adjusted to get a bright image without saturation. The transfer function of the acquisition system from the light intensity (I_light_) on the camera sensor to the pixel value (PV) in the video frame was proportional (linear with no offset: PV = G * I_light_), so that different gain settings G do not influence the relative light intensity changes.

### 2.2. Data acquisition and evaluation

Video sequences of the optic nerve head (ONH) for 7 different wavelengths (475, 501, 551, 576, 626, 650, and 677 nm, FWHM 15 nm) are acquired one after the other from one subject within a few minutes. The pupil was dilated using Mydriatikum Stulln (Pharma Stulln GmbH, Germany). Before each acquisition, the filter for the selected wavelength was inserted in the beam path and the intensity in the pupil plane was adjusted to 12 μW by changing the LED current and by inserting neutral density filter if necessary. The duration of the acquired video sequences is 8 s (200 frames) for each wavelength. Acquired video sequences are registered offline to compensate for eye movements using a two-step process (10). From registered video sequences, heart beat induced intensity changes I_raw_(n) (n: frame number) can be measured in any user defined area of interest (AOI).

To compensate for slow intensity changes that are not due to changing reflection of the retina (e.g., changing alignment of the instrument relative to the entrance pupil of the eye due to small head movements) the raw signal I_raw_(n) has to be corrected using the trend signal (moving average) I_avg_(n):


$$I_{avg}\left(n\right)=\frac{1}{25}\overset{i=n+12}{\underset{i=n-12}{\sum }}I_{raw}\left(i\right)$$

The final signal I_pleth_(n) is calculated as:


$$I_{pleth}\left(n\right)=\frac{I_{raw}\left(n\right)}{I_{avg}\left(n\right)}$$

This corresponds to a photo-plethysmografic signal. In order to adapt the display of the results to the known behavior of the heart rhythm signal, the y-axis of the plethysmography signal is inverted so that increasing blood volume (decreasing light intensity) leads in an increase of the signal (and vice versa). For one entire cardiac cycle, the pulsatile absorption amplitude (PAA) can be calculated from the plethysmography signal I_pleth_(n):


$$PAA=\frac{I_{pleth\_\max}-I_{pleth\_\min}}{I_{pleth\_\max}}$$

with I_pleth_max_ image intensity at the beginning of systole and I_pleth_min_ image intensity just after systole. A detailed description how to calculate the PAA from video sequences is given in Tornow et al. (7).

**Figure 2:**
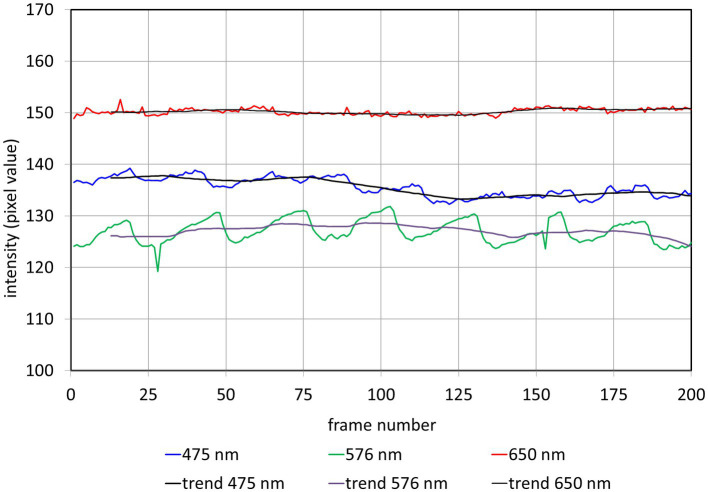
Fundus images of the same subject for different wavelengths. The images show the average image of the entire registered video sequence. The white circle shows the AOI were the PAA was taken from (see Figure 3).

Here, we use small AOIs located in the area of the ONH without visible vessels (see Figure 2) to calculate PAA for different wavelengths.

### 2.3. Absorption of a thin blood layer

The changing absorption reflects the changing blood volume in the tissue. To estimate the thickness of an equivalent blood layer, the absorption A(λ) of a thin layer of blood is calculated as A(λ) = μ_eff_(λ)*d_equi_ with μ_eff_(λ): effective attenuation coefficient and d_equi_: thickness of the equivalent blood layer. Equivalent blood layer means a layer of blood that has the same absorption as the changing fraction of blood in the ocular fundus at its maximum.

The effective attenuation coefficient μ_eff_(λ) of blood (including absorption and scattering) was calculated using the absorption and scattering coefficients and the scattering anisotropy coefficient according to Bosschaart et al. (11). The value of the thickness d_equi_ of the equivalent blood layer is varied to adjust the absorption of the blood layer to the measured PAA values.

## 3. Results

Figure 2 shows the fundus images (average image of the entire registered video sequence) of the same subject for 7 different wavelengths. The white circles show the position of the AOI where the PAA is determined.

**Figure 3:**
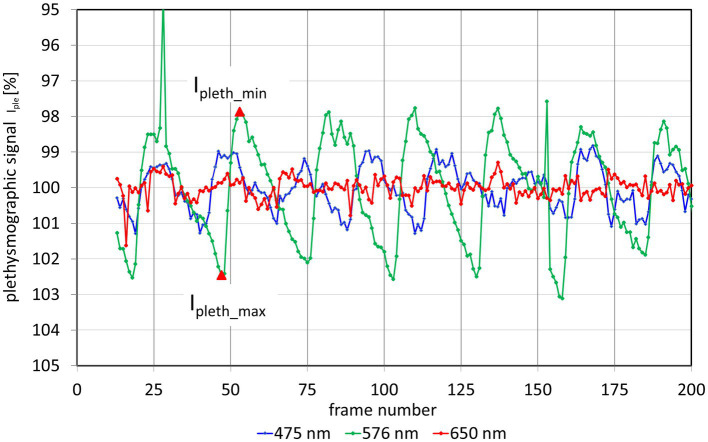
Intensity I_raw_(𝑛) and trend I_avg_(𝑛) in the selected AOI (see Figure 2) for different wavelengths. n: frame number.

**Figure 4:**
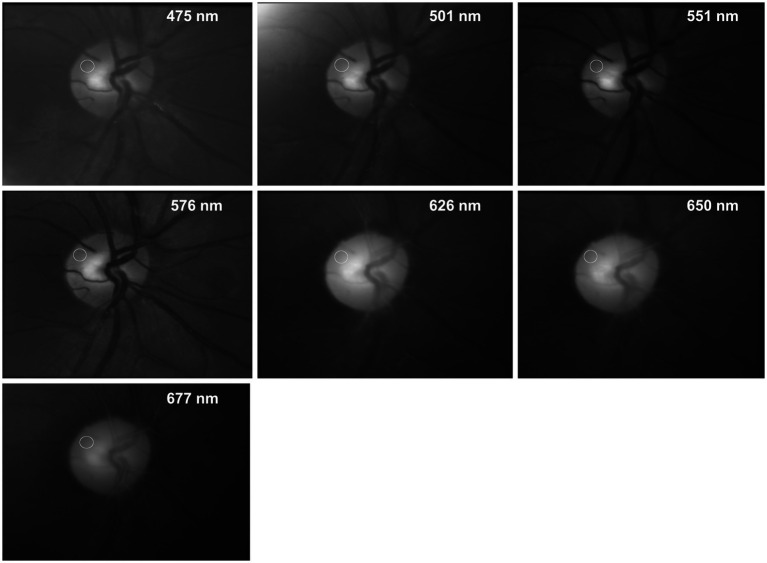
Plethysmographic signal I_pleth_ for 3 different wavelength. The y-axis of the plethysmography signal is inverted so that increasing blood volume (decreasing light intensity) results in an increase of the signal (and vice versa). The outliers in the 576 nm signal (at frames 28 and 152) are due to fast eye movements and blinks. From these plethysmographic signals the pulsatile absorption amplitude PAA can be calculated for each wavelength. As an example, the red triangles show the values I_pleth_min_ and I_pleth_max_ for the second heartbeat of the 576 nm signal.

The changing light intensity for 3 wavelengths within these AOIs is shown in Figure 3. The colored curves (blue 475 nm, green 576 nm, and red 650 nm) show the raw signal I_raw_(n) and the gray lines the corresponding calculated trend signal I_avg_(n). The plethysmographic signal after trend correction is shown in Figure 4. The green signal (576 nm) shows the highest amplitude, while for the red signal (650 nm) no pulsatile signal is visible.

**Figure 5:**
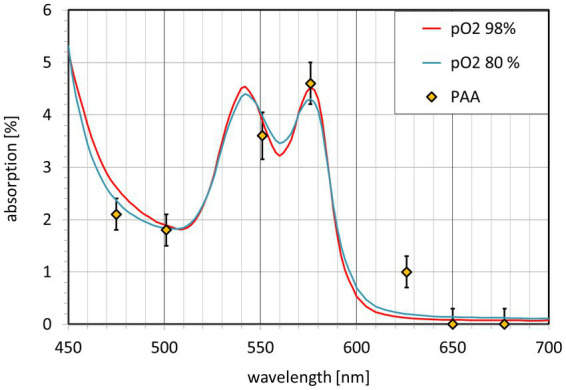
Pulsatile absorption amplitude PAA(λ) (diamonds) and light absorption A(λ) of an equivalent blood layer. The blood absorption A(λ) is shown for O_2_ saturation greater 98% (red) and 80% (blue).

The calculated PAA values for all 7 wavelengths are shown in Figure 5. PAA ranges from close to 0% at 650 nm and 677 nm to the maximum value of 4.5% at 576 nm. The intermediate values follow the theoretical light absorption of blood in the range between 475 nm and 677 nm. Additionally, Figure 5 shows the spectral distribution of an equivalent blood layer. The best fit (except for the 656 nm PAA value) is achieved for d_equi_ = 1 μm. As the light that is reflected to the CMOS camera passes the retinal tissue twice, this corresponds to an equivalent layer of about 0.5 μm thickness. The PAA value for 626 nm lies above the calculated absorption of a 0.5 μm thick blood layer.

## 4. Discussion

This key experiment shows that the mono-color video-ophthalmoscope can be extended to a multi-color instrument. In a first experiment, it is shown that the wavelength dependency of the pulsatile absorption amplitude PAA(λ) follows the spectral distribution of the light absorption of blood. This confirms that the pulsatile light intensity changes are really caused by light absorption of changing blood volume and are not due to mechanical changes of the eyeball caused by the pulsating intraocular pressure.

The absorption corresponds to an equivalent blood layer with a thickness in the range of 0.5 μm. The equivalent thickness d_equi_ for the absorption for PAA at 656 nm is higher, it is 5 μm (single path) or 2.5 μm (double path). The reason for this is unclear. Further work is necessary to develop a model for the light reflection in this area and to confirm the results.

The actual light absorption depends on blood parameters, especially on the scattering. Here, we use the values of Bosschaart et al. (11). However, other values might also apply and would change the thickness of the equivalent blood layer. For comparison, the amplitude of the cardiac cycle induced mechanical movement of the ONH tissue is in the range between about 7 μm (12) to about 13 μm (13).

Another important result is that the highest PAA is reached at about 576 nm. This value is close to the theoretically selected wavelength of the mono-color video-ophthalmoscope used in our previous clinical studies. High PAA values in normal subjects makes it easier to detect a reduction in amplitude in patients with glaucoma or other eye diseases.

Advantage of this method to measure the blood light absorption in the human retina is the fact that only components pulsating according to the cardiac cycle contribute to the PAA value. The influence of the spectral distribution of any other tissue or pigments (macula pigment, photoreceptors etc.,) is negligible, because it is not pulsating. However, it must be clearly stated, that no reliable clinical conclusions can be drawn from these first results. Further work is necessary, in the technical area, the field of data acquisition and evaluation and the interpretation of the data.

There are a lot of possibilities for technical improvements. First of all, it would be useful to add more acquisition wavelengths. Especially for blood absorption measurements, the selected wavelength can be optimized or adapted to the wavelength used for oxygen saturation measurements. For clinical applications, fast automatic switching between preselected wavelengths could be useful. This could be realized with individual light sources for each wavelength (LED and filter), so that each wavelength can be electronically switched quickly (5). Other light sources are also possible, e.g., the use of a fiber coupled light source. More than the blood absorption measurement, there will be many other applications for a multi-color video-ophthalmoscope, e.g., the measurement of oxygen saturation and a better discrimination of arteries and veins for vessel investigations.

As consequence, the development of a multi-color video-ophthalmoscope for clinical use appears possible and reasonable. One long term aim is to use two optimized video-ophthalmoscopes to measure both eyes simultaneously with exact synchronization (7) to compare both eyes to detect deviation from symmetry even in dynamic processes (e.g., pulse rise time, temporal shift between both sides) as a sign for deviation from normal behavior.

## Data availability statement

The raw data supporting the conclusions of this article will be made available by the authors, without undue reservation.

## Ethics statement

The studies involving human participants were reviewed and approved by Ethik-Kommission, Friedrich-Alexander-Universität Erlangen-Nürnberg, MedizinischeFakultät, 91054 Erlangen Germany. The patients/participants provided their written informed consent to participate in this study.

## Author contributions

R-PT: conceptualization, data acquisition and curation, data processing and analysis, manuscript drafting, methodology, project administration, and supervision. JO: image processing and manuscript revision. RK: image processing and analysis, manuscript revision, and methodology. All authors contributed to the article and approved the submitted version.

## Funding

This was supported by the Deutsche Forschungsgemeinschaft DFG (TO 115/3–1) to R-PT and Czech Science Foundation, project no. 21-18578S.

## Conflict of interest

The authors declare that the research was conducted in the absence of any commercial or financial relationships that could be construed as a potential conflict of interest.

## Publisher’s note

All claims expressed in this article are solely those of the authors and do not necessarily represent those of their affiliated organizations, or those of the publisher, the editors and the reviewers. Any product that may be evaluated in this article, or claim that may be made by its manufacturer, is not guaranteed or endorsed by the publisher.
